# Development of a laser capture microscope-based single-cell-type proteomics tool for studying proteomes of individual cell layers of plant roots

**DOI:** 10.1038/hortres.2016.26

**Published:** 2016-06-01

**Authors:** Yingde Zhu, Hui Li, Sarabjit Bhatti, Suping Zhou, Yong Yang, Tara Fish, Theodore W Thannhauser

**Affiliations:** 1 Department of Agricultural and Environmental Sciences, College of Agriculture, Human and Natural Sciences, Tennessee State University, Nashville, TN 37209, USA; 2 R. W. Holley Center for Agriculture and Health, USDA-ARS, 538 Tower Road, New York, NY 14853 Ithaca, New York, USA

## Abstract

Single-cell-type proteomics provides the capability to revealing the genomic and proteomics information at cell-level resolution. However, the methodology for this type of research has not been well-developed. This paper reports developing a workflow of laser capture microdissection (LCM) followed by gel-liquid chromatography-tandem mass spectrometry (GeLC-MS/MS)-based proteomics analysis for the identification of proteomes contained in individual cell layers of tomato roots. Thin-sections (~10-μm thick, 10 sections per root tip) were prepared for root tips of tomato germinating seedlings. Epidermal and cortical cells (5000–7000 cells per tissue type) were isolated under a LCM microscope. Proteins were isolated and then separated by SDS–polyacrylamide gel electrophoresis followed by in-gel-tryptic digestion. The MS and MS/MS spectra generated using nanoLC-MS/MS analysis of the tryptic peptides were searched against ITAG2.4 tomato protein database to identify proteins contained in each single-cell-type sample. Based on the biological functions, proteins with proven functions in root hair development were identified in epidermal cells but not in the cortical cells. Several of these proteins were found in Al-treated roots only. The results demonstrated that the cell-type-specific proteome is relevant for tissue-specific functions in tomato roots. Increasing the coverage of proteomes and reducing the inevitable cross-contamination from adjacent cell layers, in both vertical and cross directions when cells are isolated from slides prepared using intact root tips, are the major challenges using the technology in proteomics analysis of plant roots.

## Introduction

The structure of a typical root is organized from the outermost to the innermost rings as: epidermis, cortex, endodermis, pericycle and the stele tissues. The root epidermis, endodermis and pericyle are each formed of a single layer of cells, whereas the cortex comprises one to several cellular layers. In *Arabidopsis thaliana,* roots contain a single-layer cortex and tomato (*Solanum lycoperisicum*) roots have three layers.^1^ Each of the root tissue layers is comprised of a unique cell population, demonstrating different degrees of morphological, as well as functional specialization.^2^

Due to the spatial distribution of the layers, suboptimal soil conditions impart varying degrees of effects on root cells. These cellular layers also play different roles in affecting the root architectural system and functions therein. For instance, when plants are subjected to Al toxicity, distortion of the dynamics of microtubes occurred in epidermal and outer cortical cells, but not in the tissues located more centripetally in the roots.^3^ A more recent freeze–thaw experiment showed that Al-treated roots had more damage in the epidermal and outer cortex cells due to binding of Al to the cell wall and Al-induced oxidative cellular damage.^4^ Similar phenomena of positional effects of root cellular layers are also observed under osmotic, drought and salt stress treatments.^5,6^ Thus a single-cell-type analysis approach is preferred to effectively reveal the underlying molecular mechanisms regulating root developmental processes and plasticity when grown under suboptimal conditions.

Single-cell-type proteomics can provide the capability to revealing the genomic and proteomics information at cell-level resolution. The identification of cell-type specific proteins and cellular events would provide novel insights into the molecular networks and dynamics underlying the functions of specific types of plant cells.^7^ Thus far, in plants single-cell-type proteomics has been mainly applied to study cell populations that are well-separated from other tissues, such as root hairs, trichomes, cotton fiber and male and female gametes,^8–10^ and using cell suspension cultures.^7^ The recent development of the Meselect method has aided in the identification of proteomes contained in individual types of leaf cells.^11^

Laser capture microdissection (LCM) is a technique by which individual cells can be harvested from tissue sections by tacking selected cells to an adhesive film with a laser beam while they are viewed under microscope. DNAs, RNAs and proteins from individual cell types can be analyzed for genomic characteristics, and relationship between gene expression and cell-specific functions.^8,12,13^

For any specific cell type within complex organs and tissues of plants, cells collected via LCM have been largely used for DNA and RNA analysis, as the current analytical platform utilizes amplification of these nucleic acids to produce sufficient quantities of materials for downstream high-throughput analysis (such as sequencing). For instance, Matas *et al.*^14^ reported that RNA extracted from ~400 cells of tomato fruit tissue and subjected to two rounds of amplification resulted in 35–70 μg of amplified RNA per tissue sample. The sequencing reads of the RNAs were assembled into 20 976 high-quality unigenes.

Proteomics analysis of LCM tissues would require a much larger number of cells. A study on root pericycle cells of maize (*Zea mays*) indicates that ~200 cells are in each ring of pericycle circle (10 μm in thickness), and a sample of 1000 rings containing about 200 000 cells can yield 30 μg of proteins.^15^ To pick such a large number of cells would be a very costly procedure in terms of LCM instrumentation time. Some of these challenges may be reduced by increasing the mass spectrometry sensitivity with the newest model of mass spectrometry systems.

The aim of this study was to develop a workflow process for single-cell-type proteomics, and to determine if cell population-specific protein profiling could relate protein expression with function and structure that are unique for the distinct cellular layers of tomato roots. Results revealed considerable positional variation in proteome composition among those spatially distinct, but adjacent, tissues containing cell types showing clear evidence of functional specialization, such as those for root hair development in the epidermis. Data from this study has shown that coupling of LCM with the proteomics analysis can provide numerous insights into root cell-specific regulatory and metabolic pathways.

## Materials and methods

### The experimental workflow

**Figure 1:**
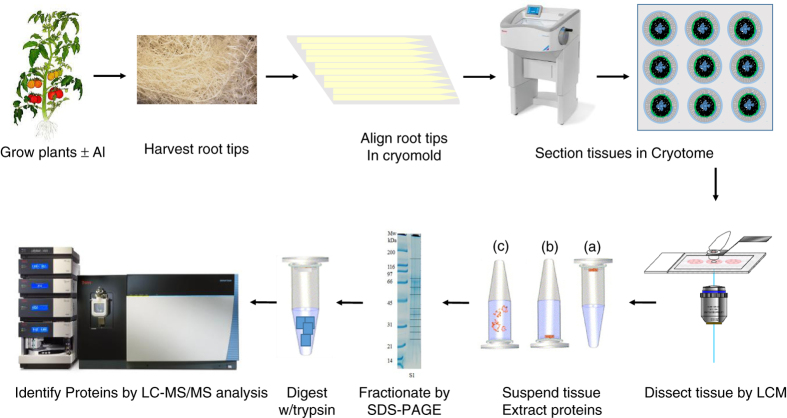
Workflow of laser capture microdissection single-cell-type proteomics of tomato roots. During step 1, roots were harvested, fixed and embedded in optimal cutting temperature compound (OCT embedding compound). Tissue sections (10 μm in thickness) were cut using a cryostat microtome at −20 °C. During step 2, using a PALM MicroBeam Laser Capture Microdissection (LCM) system, single-cell layer tissues were cut, lifted from the slides and collected into a capture tube using an ultraviolet laser. During step 3, proteins were extracted from the LCM tissues and separated on SDS–PAGE gel (1D), followed by in-gel trypsin digestion (note that the dark lines in the right lane of the electropherogram represent fraction boundaries and not distinct proteins). During step 4, the tryptic peptides were analyzed using nano liquid chromatography-tandem mass spectrometry (LC-MS/MS) and proteins were identified by comparing these spectra against theoretical spectra generated *in silico* from the unigenes in ITAG2.4 tomato protein database. 1D, one-dimensional; SDS–PAGE, SDS–polyacrylamide gel electrophoresis.

The process of single-cell-type proteomics consists of four steps (Figure 1): (1) Preparation of thin-sectioning of root tips; (2) LCM and single-cell-type tissue collection; (3) Protein extraction and processing; and (4) Nanoflow liquid chromatography-tandem mass spectrometry (LC-MS/MS) and database search for protein identification. The experimental procedure in this study followed this workflow. Finally, the relationship between the identified proteins and cell functions was determined using information available in public databases and text mining of published materials.

### Plant material preparation and Al treatments

We chose tomato roots because tomato is sensitive to aluminum toxicity and the cell-layer proteins could be evaluated. As described above, the epidermal and cortical, as well the inner layer cells of tomato roots would respond differentially to Al treatments.^3,4^ Tomato ‘Micro-Tom’ seeds were surface disinfected by soaking in 20% commercial bleach for 20 min, followed by three washes in distilled water. Seeds were sown into rockwool blocks, which were submerged in Magnavaca’s hydroponic solution, pH 4.5.^16^ The strength of the Al treatment was kept at 14.5 μM Al^3+^ activity (by adding 100 μM AlK(SO_4_)_2_·12H_2_O).^17^ Seedlings were harvested 10 days after seed germination when the two cotyledons had enlarged and lateral roots were seen on some Al-treated plants. About 2000 seeds each for Al-treated and non-Al-treated experiments were germinated in three hydroponic tanks. Initially, the three tanks were designed as biological replicates. However, at the end of experiment, less than half of the seeds in Al-treated tanks produced uniform-sized seedlings. To ensure a reasonable sample size for each treatment experiment, these plants were pooled into one sample during the following analysis.

### Tissue preparation for LCM harvest

Preparation of root tips for LCM followed the protocol described by Matas *et al.*^14^ with modifications for root tissues. Radicles attached to a short segment of hypocotyl (the thicker hypocotyl ends make it easier to handle during the optimum cutting temperature (OCT) embedding process) were cut off the seedlings immediately after they were lifted out of the hydroponic tank. Collected tissues were submerged in a fixative solution containing 75% (v/v) ethanol and 25% (v/v) acetic acid, at a 1:10 volume ratio of tissue to fixative on ice. Fixative was infiltrated into the tissue under vacuum for 15 min on ice and then replaced with fresh solution, before incubating at 4 °C overnight. Tissues were transferred to 10% (w/v) sucrose in phosphate-buffered saline (137 mM NaCl, 8 mM Na_2_HPO_4_, 2.7 mM KCl, and 1.5 mM KH_2_PO_4_, pH 7.3) and protease inhibitor (1:100 volume ratio dilution) (Sigma, St Louis, MO, USA). After infiltration for 15 min, buffer was then exchanged for 20% (w/v) sucrose in the same phosphate-buffered saline and protease inhibitor buffer, and the infiltration step was repeated as before. Tissues were washed in OCT medium (VWR, Radnor, PA, USA), and transferred to intermediate (10×10×5 mm) cryo-molds filled with OCT, and frozen in a glass beaker chilled over liquid nitrogen. Root tips were aligned to one end of the cryomold, and about 50 root tips were placed into each block. The frozen blocks were transferred to larger cryomold (22×22 mm), and frozen following the same procedure. The frozen tissue blocks were placed on dry ice for immediate micro-sectioning, or stored at −80 °C.

### Laser capture microdissection

Root-tip tissues were sectioned at a thickness of 10 μm in a cryostat (Microm HM550; Thermo Fisher Scientific, Grand Island, NY, USA), and mounted on Arcturus polyethylene naphthalate (PEN) Membrane Slides (Thermo Fisher Scientific) at −20 °C. Ten root-tip sections were collected for each frozen block. Frozen sections were placed on dry ice and were immediately used for LCM, otherwise they were stored at −80 °C. The LCM procedure was conducted using a PALM Laser Microbeam instrument (Carl-Zeiss, Oberkochen, Germany). The frozen slides were dipped in 100% ethanol for 1 min before LCM to remove all the moisture. During the LCM process, the same individual cell-layer tissues were collected at one time, and then the slides were stored at −80 °C until collection of the next cellular layer samples.

### Protein extraction from capture caps

Proteins from the isolated cells were extracted using a modification of a previously reported method.^18^ Each LCM sample capture tube (0.5 mL in size) was filled with 200 μL of a dense SDS buffer containing 0.5 M Tris-HCl, pH 7.5, 50 mM EDTA, 0.1 M KCl, 0.7 M sucrose, 2% (w/v) SDS, 2% (v/v) 2-mercaptoethanol and proteinase inhibitor cocktail (Sigma). The tube was placed on a rotary shaker in a cold room overnight (−4 °C). Then a clean pipette tip was used to tease the LCM tissues together with the opaque adhesive material out of the capture cap, which was then transferred into a pre-chilled mortar (using liquid N_2_). After transferring the extraction buffer into the mortar, sample was ground into a fine powder under liquid N_2_. Another 100 μL extraction buffer was used to rinse proteins off the grinding utensils. The tissue and buffer mixture was transferred into 2 mL Eppendorf tubes. After adding an equal volume of saturated phenol (pH 8.0), the mixture was vortexed briefly and placed on ice for 1 h, vortexing at 10-min intervals. After centrifugation at 4 °C for 15 min at 16 000 *g*, the supernatant was transferred to a clean tube, incubated overnight at −20 °C and proteins were precipitated by adding 4 vol of 0.1 M ammonium acetate in methanol. Protein pellets were collected after centrifugation at 4 °C for 20 min, dissolved in a protein dissolution buffer containing 8 M urea, 2 M thiourea and 2% CHAPS (3-[(3-cholamidopropyl) dimethylammonio]-1-propanesulfonate).^19^ Proteins were concentrated using 5 K spin ultrafiltration devices (EMD Millipore, Billerica, MA, USA) by centrifugation at 16 000 *g* until the volume for each sample was reduced to about 20–30 μL. Protein concentration was estimated visually based on color changes by adding 1 μL of protein in 50 μL protein assay buffer. Bovine serum albumin was used to prepare the protein concentration standard (The Bio-Safe Coomassie, Biorad, CA, USA). As the protein sample was very small, no replicate was conducted, nor was the protein concentration measured on a spectrometer which would have consumed a large portion of the protein sample.

Proteins were separated on a 10–20% gradient Tris-glycine minigel followed by Colloidal Coomassie blue staining. Each lane containing proteins from a single sample, was divided into 5–11 fractions for in-gel trypsin digestion.^19,20^ All the samples were stored at −20 °C until analysis.

### Proteomics analysis

Proteins were identified using either an Orbitrap Elite spectrometer or Fusion mass spectrometer (Thermo Fisher Scientific, San Jose, CA, USA). The analyses carried out on the Elite (the cortical samples) involved the serial analysis of 10 or 11 individually digested gel fractions. Those carried out on the Fusion (the epidermal samples) consisted of a single injection of a sample created by pooling the five separately digested gel fractions as the more rapid scanning rate of the Fusion minimized the need for pre-fractionation. The mass data obtained was used to interrogate the tomato protein database, iTAG2.4, to obtain protein identifications.

### Nano LC-MS/MS

Each of the one-dimensional gel band digestion was reconstituted in 30 μL of 2% acetonitrile (ACN)/1% formic acid (FA) for nano LC-MS/MS analysis. The investigation of the cortical samples involved the serial injection of these individually digested gel band fractions on a nano scale liquid chromatograph (described below), which was connected to an Orbitrap Elite mass spectrometer equipped with a nano ion source using collision-induced dissociation (CID) as described below. The investigation of the epidermal samples comprised a single injection of a pool of the five individually digested gel bands on a nanoscale liquid chromatograph linked to an Orbitrap Fusion Tribrid (Thermo Fisher Scientific) mass spectrometer similarly equipped with a nano ion source. This sample was created by pooling the individual 30-μL aliquots of the reconstituted in-gel band fractions and drying it under reduced pressure. The dried, pooled sample was then re-dissolved in 30 μL of 2% ACN/1% FA.

Both the mass spectrometers were coupled with an UltiMate3000 RSLCnano (Dionex, Sunnyvale, CA, USA) and used the same LC method. Each sample (15 μL) was injected onto a PepMap C-18 reversed-phase (RP) nano trap column (3 μm, 75 μm×20 mm, Dionex) with nanoViper Fittings at 20 μL min^−1^ flow rate for on-line desalting and then separated on a PepMap C-18 RP nano column (3 μm, 75 μm×15 cm), and eluted in a 60-min gradient of 7–38% ACN in 0.1% FA at 300 nL min^−1^, followed by a 5-min ramp to 95% ACN/0.1% FA and a 7-min hold at 95% ACN/0.1% FA. The column was re-equilibrated with 2% ACN/0.1% FA for 20 min prior to the next run. The Orbitrap Elite was operated in positive ion mode with nano spray voltage set at 1.6 kV and source temperature at 275 °C. The instrument was externally calibrated using Ultramark 1621 (Thermo Fisher Scientific, San Jose, CA, USA) for the Fourier transform-based mass analyzer. An internal calibration was performed using the background polysiloxane ion signal at a mass-to-charge ratio (*m/z*) of 445.120025 as the calibrant. The instrument was operated in data-dependent acquisition (DDA) mode. In all experiments, full MS scans were acquired over a mass range of 400–1400 *m/z*, with detection in the Orbitrap mass analyzer at a resolution setting of 120 000. Fragment ion spectra produced via CID were acquired in the Orbitrap mass analyzer. In each cycle of DDA analysis, following each survey scan, the 15 most intense multiply-charged ions above a threshold ion count of 5000 were selected for fragmentation at normalized collision energy of 35%, and for the Orbitrap Fusion the instrument was calibrated in a similar manner. Each precursor ion scan was followed by a 3-s ‘Top Speed’ data-dependent CID ion trap MS/MS with a 1.6 *m/z* window for quadrupole isolation of precursor peptides with multiply-charged ions with intensities above a threshold ion count of 10 000 using normalized collision energy of 30%. Dynamic exclusion parameters were set at repeat count 1 with a 30-s repeat duration, an exclusion list size of 500 ions and a 60-s exclusion duration with ±10 p.p.m. exclusion mass width. All data were acquired with Xcalibur 2.2 software (Thermo Fisher Scientific, San Jose, CA, USA).

### Data processing, protein identification and data analysis

All MS and MS/MS raw spectra were processed using Proteome Discoverer 1.4 (PD1.4, Thermo Fisher Scientific, San Jose, CA, USA) and the spectra from each DDA file are output as an MGF file for subsequent database search using in-house licensed Mascot Daemon (version 2.5.1, Matrix Science, Boston, MA, USA). The ITAG2.4 tomato proteins database^21^ containing 34 973 sequence entries was downloaded on July 2015 from http://solgenomics.net/tomato/ and used for database searches. The default search settings used for the Mascot analysis were: one missed cleavage site by trypsin allowed with fixed Methylthio modification of cysteine, and variable of oxidation on methionine and deamination of Asn and Gln residues. The peptide and fragment mass tolerance values were 10 p.p.m. and 0.8 Da, respectively. To reduce the probability of false identification, only peptides with significance scores at the 99% confidence interval were counted as identified.^22^

### Functional pathways prediction and spatial distributions

Functional pathways were analyzed using the MapMan tools (Version 3.6.0RC).^23,24^ Proteins annotated to root hair development were searched in the root hair genomics database (iRootHair).^25^ Proteins of unigenes annotated to epidermis, cortex and Al stress were also searched in the annotated tomato database.^21^ In addition, literature search was also used for the identification of protein functions relevant to root hair traits and Al stress.

## Results and discussions

### LCM tissue harvest and extraction of proteins from single-cell-type samples

**Figure 2:**
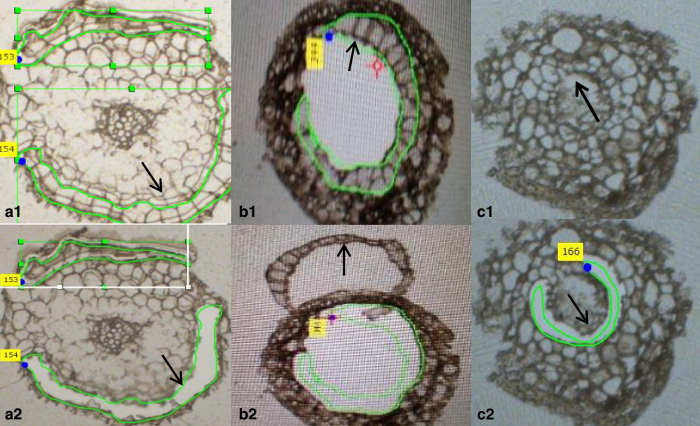
Harvesting of single cellular layer tissues from tomato roots using laser capture microdissection. The epidermal (**a1** and **a2**, before and after LCM), cortical (**b1** and **b2** after LCM) and endodermal (**c1** and **c2**, before and after LCM) cellular layers were isolated following the LCM procedure as described in ‘Materials and Methods'. LCM, laser capture microscope.

The root sections (10 μm in thickness) were transferred to PEN-frame slides. When freshly prepared sections were washed in 70% ethanol, the tissues tended to become loose and fall off the membrane. Also, there was a concern about potential protein loss during serial washes of the slides in 70–85% ethanol which are used to remove OCT solution from the slides. Thus, repetitive washing steps were omitted. Instead, all the slides were dipped in 100% ethanol for 1 min to remove the moisture which is known to interfere with the LCM procedure. On some slides, the anatomical structures of the cross sections were covered by OCT solution (Figures 2a1–b2), but the epidermal and cortical cell layers were still clearly defined which allowed for collection of the two distinct types of tissues. On some slides, cell layers on the root cross-section are very distinct, and the endodermal cellular layers were harvested from these slides (Figures 2c1 and c2). In this study, about 5000–7000 cells were collected for the epidermal and cortical layers from 300 to 500 sections. As only a small number of slides were amenable for the LCM of endodermal cells, a very small-sized sample was produced which was thus excluded from further analysis.

To recover sufficient amounts of proteins from a very small amount of starting materials at the milligram scale is a very challenging task.^26^ It is particularly true for the LCM sample from plant tissues. Therefore, in this study a great amount of effort was expended to optimize the protein extraction protocol. To release proteins from plant cells, tissue homogenization is a critical step and can only be achieved using mechanical methods. No cell lysis enzymes can be used as they will interfere with the downstream proteomics analysis. In this study, we used the conventional mortar and pestle method to extract protein. The use of this method incurs the risk of losing proteins that stick onto the utensils. Repeated washes lead to a large volume of extraction buffer, which can reduce the recovery efficiency of protein during precipitation. In this study, all the utensils were washed three times, buffer for each wash was collected in separate tubes, and proteins were precipitated accordingly. Samples were combined into one tube after protein pellets were solubilized.

**Figure 3:**
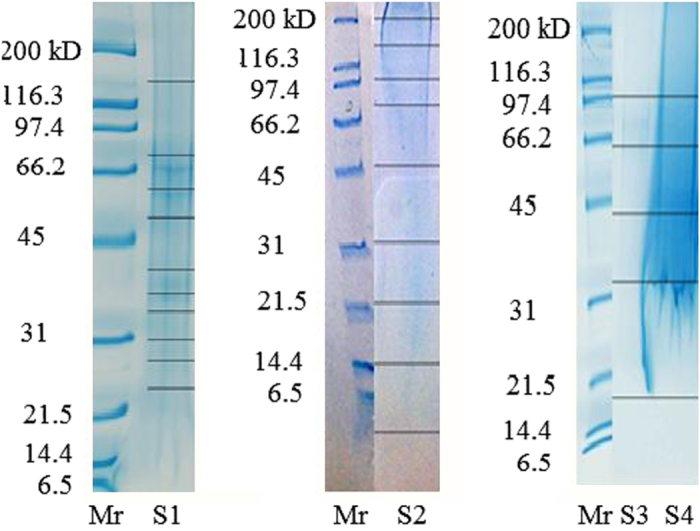
SDS–PAGE gel images of proteins extracted from epidermal and cortical cells of tomato roots. Lane S1, Al-treated cortical protein; lane S2, non-Al-treated cortical protein, lane S3, Al-treated epidermal protein; lane S4, non-Al-treated epidermal protein.

When using minigels for protein electrophoresis, each well allows for the loading of up to 40 μL of sample. Taking into consideration the volume of the loading buffer, only a maximum of 30 μL of protein samples can be loaded onto the gel. In this study, all the protein samples exceeded this volume after completely dissolving all the pellets. There are different methods for reducing the volume of protein extracts, such as the trichloroacetic acid–acetone precipitation or similar methods which all result in up to 20–50% loss of proteins.^19,20,27,28^ In this study, we first used the vacuum drying method to reduce the volume of the samples. The concentrated proteins formed smeared bands on the SDS–PAGE gel (Figure 3. Supplementary Figure S2). The second approach used the centrifugal ultrafiltration device, which seems more effective as it was able to remove larger volume of buffer resulting in a higher protein concentration (Figure 3 and Supplementary Figure S4). However, none of the protein samples were separated into clear bands on SDS–PAGE gel. The smeared protein gel could be caused by protein sample overloading, which occurs for protein separation on SDS–PAGE gel electrophoresis.^29^ In addition, the high-urea content in the protein dissolution buffer may also have interfered with the protein separation process. It is very obvious that this protein extraction and reconstitution method needs to be improved.

### Identification of single-cell-type proteomes of tomato roots:

As shown in Figure 3, a majority of the gel pieces did not have clear bands. However, proteins were identified in each of the tryptic digest fractions. In total, 1313 proteins were detected in the Al-treated epidermal cell population, 744 proteins in the Al-treated cortical cell population, 365 proteins in the non-Al-treated epidermal cell population and 745 proteins in the non-Al-treated cortical cell population. Between Al-treated epidermal and cortical proteomes, 543 proteins overlapped in these two layers of tissues. Between the non-treated epidermal and cortical proteomes, 189 proteins overlapped in these two types of tissues. In both cases, a significant portion of proteome contained in cells from different layers of tissues in roots was composed of proteins unique to the individual cell types.

**Figure 4:**
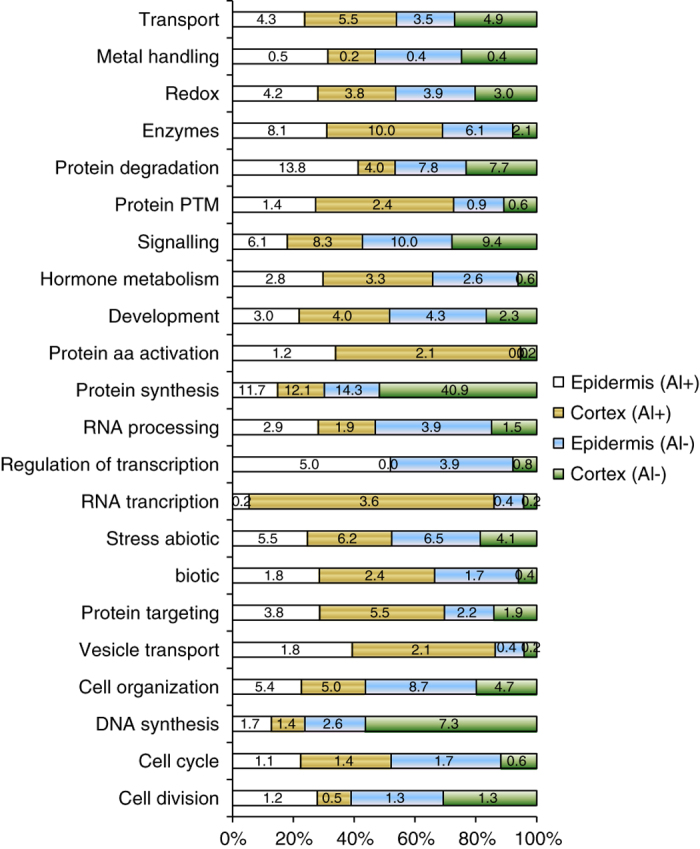
Distribution of proteins among functional pathways identified in single-cell-type proteomes of tomato roots. Proteins identified in each tissue sample were analyzed for functional pathways using the MapMan tools.^24^ The numbers are the percentage of proteins in the pathway of the total proteome identified in the respective tissue. Al+, Al-treated; Al−, non-Al-treated tissues.

As shown in Figure 4 and Supplementary Table S1, the functional pathway analysis indicates that the Al-treated epidermal cells contained the highest percentage of proteins in protein degradation (13.8% compared with 7.8% in the non-Al-treated counterpart), and the lowest number of proteins in protein synthesis (11.7% compared with 14.3% in the non-Al-treated counterpart). These differences in proteome expression in Al-treated and non-Al-treated tissues might be related to the biological properties of these cells. It is known that under stress conditions, protein translation is in most cases reduced compared with normal condition, which is a major strategy for improving proteostasis.^30^

The number of proteins involved in DNA synthesis is much smaller in the Al-treated epidermal tissue (1.7%) compared with the non-Al-treated cell population (7.3%). Similarly, the Al-treated cortical cells contained the lowest percentage of proteins in the cell division pathway, DNA synthesis and protein synthesis (12.1% compared with 40.9% in non-Al-treated counterpart tissue). On the other hand, the Al-treated tissues were identified with more proteins in protein post-translational modification.

### The relationship between proteome expression and physiological and anatomical functions of single-layer cells of tomato roots

A list of proteins with a role in specified functions of epidermis and cortex in roots, and Al-related proteins are described in Table 1. First we searched the tomato database for unigenes annotated to the epidermis. This group consists of lipoxygenases, and the curculin-like (mannose-binding) lectin family proteins which are annotated as an epidermis-specific secreted protein. These proteins were identified in both the Al-treated and non-Al-treated epidermal cells.

One of the most prominent cellular activities of epidermal cells is that the trichoblasts can give rise to root hair. A large number of proteins with a role in root hair development were identified in the epidermal proteomes. In the Al-treated epidermal proteome, the DRL1 (DEFORMED ROOTS AND LEAVES 1) (solyc08g008250.1.1) and RHL1, HYP7|RHL1 (ROOT HAIRLESS 1) (solyc03g098620.2.1.) were found. The drl1 mutant shows highly abnormal development with stunted roots and few root hairs.^31^ Recessive mutants of RHL1 prevented the formation of hairs on primary roots, indicating the key role of this protein in root hair development.^32^ In the non-Al-treated control epidermal cell proteome, ROOT GROWTH DEFECTIVE 3/TATA-binding protein-associated factor MOT1 (solyc08g074520.1.1) was identified which is required for appropriate root and root hair cell enlargement.^33^ Loss-of-function mutations of ROOT HAIR DEFECTIVE3 suppress root waving, skewing and epidermal cell file rotation in *A. thaliana*.^34^

Then we searched the genes with confirmed functions in polar cell expansion and root hair tip growth in the iRootHair database, and several tomato homologous proteins were identified in the epidermal cells (Table 1). These proteins include arabinogalactan proteins (AGPs),^35^ the AGD9 (ARF-GAP DOMAIN 9), ADP-ribosylation factor GTPase-activating protein 1 (ARF1),^36^ fasciclin-like AGP10 and MEE 58 adenosylhomocysteinase 1. The tomato unigenes for cullin-associated NEDD8-dissociated protein 2 (ETA2),^21^ Villin-3 (ref. 37) and Profilin^38^ were annotated in root hair biological process. These epidermal proteins are related to root hair initiation and development from the epidermal cells of roots.

None of the proteins involved in root hair development were found in either the Al-treated or the non-Al-treated control cortical samples. Instead, the cortical myrosinase-binding protein-like protein was identified in the Al-treated cortical cells (Table 1). These results demonstrate clearly that the protein composition in each cellular layer is related directly with the cellular and physiological properties of the respective root tissue.

### Proteins for responses to Al toxicity in distinct cell layers of tomato roots:

Previous studies have used tissue-based proteomics analysis to establish the relationship between protein expression and the stress responses in tomato root tips.^18,20^ As described previously, the root tip is a complex tissue consisting of distinct cell types distributed in separate layers, each of which may be responsible for distinct aspects of an overall response mechanism. This single-cell-type proteomics study seems to be able to provide the information that is missing in the whole tissue-based analysis. This single-cell proteomics analysis has found, for the first time in tomato root proteome, several important proteins which are related to Al-induced morphological characteristics of roots. These proteins include the ROOT GROWTH DEFECTIVE 3, ROOT HAIRLESS 1 required for root hair initiation in *Arabidopsis*^32^ and DEFORMED ROOTS AND LEAVES 1 for short root phenotypes, in the epidermal proteins.

The tomato homolog for Wali7 (wheat aluminum-induced protein 7, asparagine synthetase B) protein is an Al-inducible gene first isolated in wheat (*Triticum aestivum*) roots.^39^ The LCM proteomics experimental results indicate that that this protein accumulated in epidermal and cortex cells in Al-treated roots, but only in the former layer of cells in the non-treated roots. A previous study^40^ also showed the correlation between the abundance of this protein and the specific cellular functions. It was found that asparagine synthetase was mainly localized in phloem cells of the main vascular bundles and in secondary veins of the leaf blade when tomato leaves were infected by the bacterial pathogen *Pseudomonas syringae* pv. tomato. Taken together, these results suggest that the Al-induced changes in this protein might have occurred in cortical cells, but the mechanisms remain to be elucidated.

Antioxidant enzymes play key roles in plants to reduce the cellular oxidative injury resulting from exposure to Al toxicity. However, these enzymes belong to multiple protein families each having several isoforms. Based on the single-cell-type proteomics analysis results, one unigene encoding for peroxiredoxin IIF involved in mitochondrial redox homeostasis was found only in epidermal cells. Three unigenes encoding for thioredoxin, monodehydroascorbate reductase and 2-cysteine peroxiredoxin B were found in Al-treated epidermal, as well as cortical tissues. We have also identified enzymes that were expressed in epidermal tissues from both Al-treated and non-Al-treated roots, these enzymes may have a role in maintaining redox homeostasis rather than in the antioxidant mechanism against Al stress. Catalase 2 was found in all the tissues, which is consistent with its role as a housekeeping enzyme to protect cells from the toxic effects of hydrogen peroxide.

In addition, a large number of transcription factors, proteins in cell division and cell cycle, cell signaling, cell wall modification proteins, ABC transporters and multidrug resistance systems, and other functional pathways were found in Al-treated epidermal and/or cortical cells, but not in the respective counterparts of the non-treated roots (See Supplementary Table S1). Apparently, the LCM single-cell-type proteomics has an advantage in revealing proteins that are specific to cell functions, and providing a much higher chance for the identification of low abundance proteins such as transcription factors. However, the coverage of the proteomes generated in this study using LCM single-cell-type cells, is still very low (<3% of the 33 000 predicted proteins in annotated tomato genome).

## Conclusion

In this study, we have developed a protocol for using LCM proteomics analysis of plant roots. Results demonstrate that functions of the identified proteins are correlated with morphological and physiological properties of the respective tissues. The single-cell-type proteomics analysis is particularly useful for studying the molecular mechanisms for Al toxicity and similar types of stresses that have varied effects on different layers of root cells. This claim is strongly supported by the identification of the several Al-stress-related proteins, such as metal handling proteins, ferritin and several proteins belonging to the ABC transporters and multidrug resistance systems, only in the epidermal cells, but not in the cortical cells. On the other hand, a large number of proteins were found in both epidermal and cortical cell layers, such as catalase regulating redox status as a housekeeping function. Therefore, the single-cell-type proteomics analysis greatly expands our understanding of the relationship between protein expression and biological functions of individual cells, compared with tissue-based proteomics approach.

As described in this manuscript, there is much of room for improvement in the technical procedures for protein extraction and separation, and to increase the coverage of the proteomes using the LCM approach. In future studies, the use of quantitative proteomics analysis and experiments with biological replicates will produce more convincing results. The LCM remains a very time-consuming and thus a very costly procedure for the use of this technology. As more sensitive mass spectrometry methods are developed, a smaller amount of protein for a proteomics analysis. Single-cell-type proteomics will be required a wide application in studying tissues such as roots composed of layers of cells each having distinctive structural properties and functions.

## Figures and Tables

**Table 1:** Cell- specific functions of proteins identified in single cellular layers of tomato roots^a^

*Protein accession*^b^	*Protein score*^c^	*Protein matches*^d^	*Protein coverage*	*Protein name*	*Tissue type*^e^ *Al-Ep Ct-Ep Al-Ctx Ct-Ctx*			
*Epidermal cell-expressed proteins*								
solyc07g062480.1.1	205	6	17.5	Curculin-like	v^f^	v	v	v
solyc08g014000.2.1	144	5	8.5	Lipoxygenase	v			
solyc04g054980.2.1	162	7	30.3	Lipoxygenase	v			
solyc03g093360.2.1	162	8	18.4	Lipoxygenase	v			
solyc08g029000.2.1	54	1	1.5	Lipoxygenase	v			
solyc01g099180.2.1	44	2	2.2	Lipoxygenase	v			
solyc08g014000.2.1	144	5	8.5	Lipoxygenase	v			
solyc04g054980.2.1	162	7	30.3	Lipoxygenase		v		
solyc01g099190.2.1	44	1	1.5	Lipoxygenase		v		
								
*Root hair-related proteins*								
solyc02g065740.2.1	90	3	5.6	Alpha-1 4-glucan-protein synthase	v			
solyc07g063550.2.1	34	1	1.7	Arf-GAP	v	v		
solyc09g010520.2.1	32	1	1.9	ADP-ribosylation factor	v			
solyc10g080100.1.1	97	4	4.1	Villin-3	v			
solyc12g008590.1.1	103	6	50	Profilin	v			
solyc09g092380.2.1	418	20	26.2	MEE58	v	v		
solyc01g103010.2.1	40	1	1.8	Cullin-associated protein 2	v			
solyc03g114860.2.1	104	4	8.5	Alpha-1 4-glucan-protein synthase	v			
solyc03g019750.2.1	27	1	2.9	Alpha-1 4-glucan-protein synthase	v			
solyc02g065740.2.1	90	3	5.6	Alpha-1 4-glucan-protein synthase	v			
solyc07g065540.1.1	40	1	4.4	Fasciclin-like arabinogalactan protein 10	v			
solyc08g008250.1.1	54	1	6	DEFORMED ROOTS AND LEAVES 1 (transcription factor)	v			
solyc08g074520.1.1	25	1	5.3	ROOT GROWTH DEFECTIVE 3		v		
								
*Cortex-specific proteins*								
solyc09g083020.1.1	69	1	9.6	Myrosinase-binding protein-like protein			v	
								
*Al-response proteins*								
solyc09g082780.2.1	37	1	4.4	Wali7	v	v	v	
								
*Oxidative stress*								
solyc01g079820.2.1	34	1	6.3	Peroxiredoxin IIF	v			
solyc02g078360.2.1	45	1	5.4	Thioredoxin	v		v	
solyc09g009390.2.1	112	3	10.6	Monodehydroascorbate reductase	v		v	
solyc10g082030.1.1	72	3	11.6	2-Cysteine peroxiredoxin B	v		v	
solyc09g007270.2.1	179	5	24	Ascorbate peroxidase 2	v	v	v	
solyc06g049080.2.1	69	2	6.6	Manganese superoxide dismutase 1	v	v	v	
solyc01g106450.2.1	67	3	10.6	L-galactose dehydrogenase	v	v		
solyc04g080850.2.1	35	1	8.9	Cytochrome b5	v	v		
solyc06g005150.2.1	307	11	26	Ascorbate peroxidase 1	v	v		
solyc07g020860.2.1	52	2	6.8	Thioredoxin-dependent peroxidase 1	v	v		
solyc12g094620.1.1	245	9	22.2	Catalase 2	v	v	v	v

a The functions of the proteins were identified by searching in tomato^20^ and iRootHair^24^ databases.

b Unigenes accession number in annotated tomato database iTAG2.4.

c Protein score, which are derived from the some of the peptide scores using multidimensional protein identification technology (MUDPIT) scoring.

d Peptide numbers matching the protein.

e Single cellular layers in tomato roots.

f Protein identified in the respective tissue.
